# Artificial intelligence in cardiovascular diseases: diagnostic and therapeutic perspectives

**DOI:** 10.1186/s40001-023-01065-y

**Published:** 2023-07-21

**Authors:** Xiaoyu Sun, Yuzhe Yin, Qiwei Yang, Tianqi Huo

**Affiliations:** 1 National Institute of Hospital Administration, National Health Commission, Beijing, China; 2 The Sixth Clinical Medical School, Capital Medical University, Beijing, China; 3 Department of Thorax, The First Hospital of China Medical University, Shenyang, Liaoning, China

**Keywords:** Artificial intelligence, Cardiovascular disease, Machine learning, Cardiovascular diagnosis, Cardiovascular imaging

## Abstract

Artificial intelligence (AI), the technique of extracting information from complex database using sophisticated computer algorithms, has incorporated itself in medical field. AI techniques have shown the potential to accelerate the progression of diagnosis and treatment of cardiovascular diseases (CVDs), including heart failure, atrial fibrillation, valvular heart disease, hypertrophic cardiomyopathy, congenital heart disease and so on. In clinical scenario, AI have been proved to apply well in CVD diagnosis, enhance effectiveness of auxiliary tools, disease stratification and typing, and outcome prediction. Deeply developed to capture subtle connections from massive amounts of healthcare data, recent AI algorithms are expected to handle even more complex tasks than traditional methods. The aim of this review is to introduce current applications of AI in CVDs, which may allow clinicians who have limited expertise of computer science to better understand the frontier of the subject and put AI algorithms into clinical practice.

## Introduction

As a branch of computer science, artificial intelligence (AI) is a new technical science, simulating and extending human intelligence to handle complex issues [1]. AI mimics the human brain to process data and places an essential role in medicine, which could identify, process, integrate, and analyze massive amounts of healthcare data (medical records, ultrasounds, medications, and experimental results) [2]. Specific algorithms on existing big data yield provide results that clinicians can use to improve diagnosis; for example, echocardiogram (ECG) processed by AI algorithms are currently used to diagnose heart failure [3–5], atrial fibrillation [6], anaemia [7], hypertrophic cardiomyopathy [8] and pulmonary hypertension [9]. Once validated and tested algorithms are put to work in the clinic, they could reduce clinicians’ cognitive burden by offering pre-diagnosis, correcting clinician errors and preventing the occurrence of misdiagnosis [10].

AI depends on machine learning, which could capture subtle connections from a series of data rather than manually encoding. Accordingly, these subtle findings might revolutionize the progression of human diseases in prediction, diagnosis, prognosis and recovery [11]. The subdisciplines of AI include cognitive computing, deep learning, and machine learning (ML) [12]. Machine learning is a more popular subdiscipline of AI, typically, and could be grouped into three categories: supervised learning, unsupervised learning and reinforcement learning based on the presence or absence of external supervision during training [13]. Supervised learning is the process of tuning the parameters of a classifier to achieve the required performance using a set of samples from a known class, also known as supervised training. In general, supervised learning includes artificial neural network (ANN), support vector machine (SVM), decision tree, random tree, naïve Bayes (NB), fuzzy logic, K-nearest neighbour (KNN) and regression [14]. Unsupervised learning is a data processing method that achieves the classification of samples by data analysis of a large number of samples of the object under study without category information, including clustering algorithms and association rule-learning algorithms [15]. Reinforcement learning could be considered a combination of supervised and unsupervised learning, and it could facilitate errors and trials to magnify the accuracy of algorithms [16]. The above algorithms are not completely independent, e.g., ANN can be used in the DL algorithm.

**Fig. 1:**
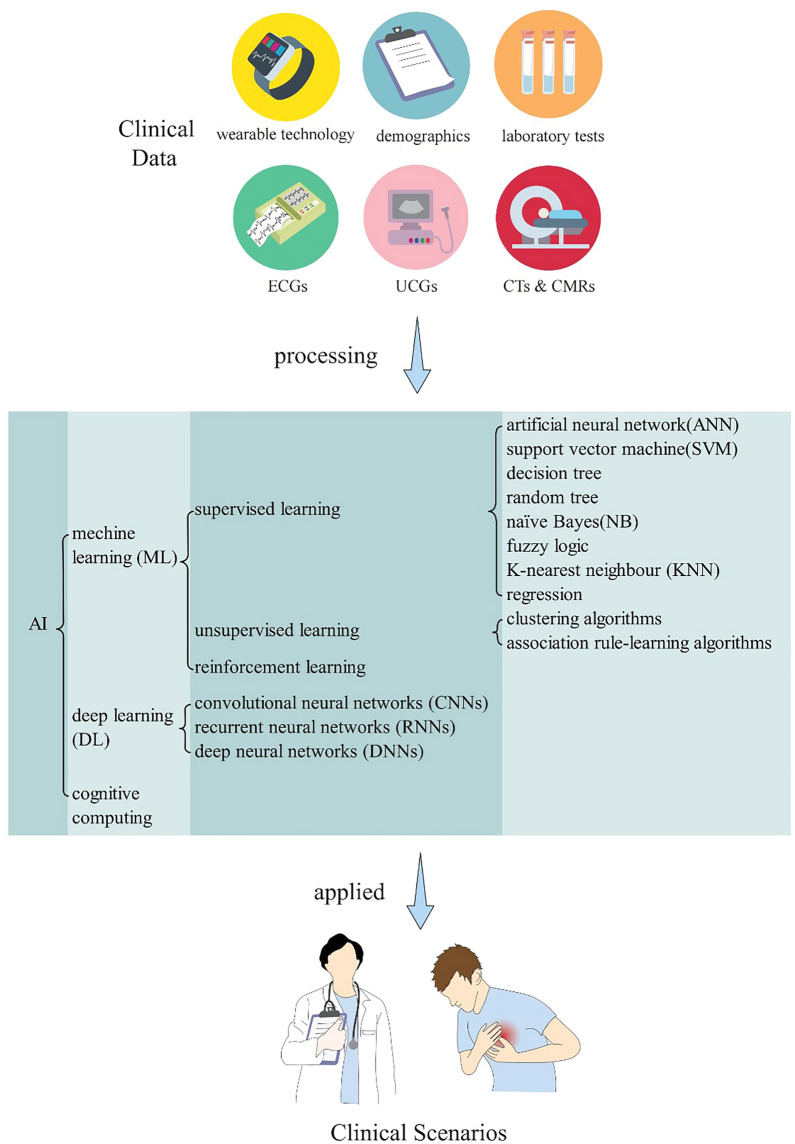
Flow chart of AI application in clinical practice

Nowadays, the most currently applied algorithms for medical purposes are deep learning (DL), artificial neural network (ANN), and support vector machines (SVM) [17]. ANNs and SVMs could high preciously deal with large and complicated data, including nonlinear relations [18]. ANNs have better superiority in assessing electrocardiogram (ECG) data [19], while SVMs are in disease stratification [20]. However, ANNs and SVMs could not dispose all conditions equally because of over-fitting, underfitting, and misspecification [21]. Deep learning has relatively good performance in processing image data, and current deep learning algorithms in cardiovascular medicine include convolutional neural networks (CNNs), recurrent neural networks (RNNs), and deep neural networks (DNNs) [22]. Even though these algorithms have corresponding advantages and disadvantages, they perform well in the diagnosis, prediction, and stratification of cardiovascular diseases (CVDs). The flow chart of the application of AI in clinical practice is shown in Fig. 1.

**Fig. 2:**
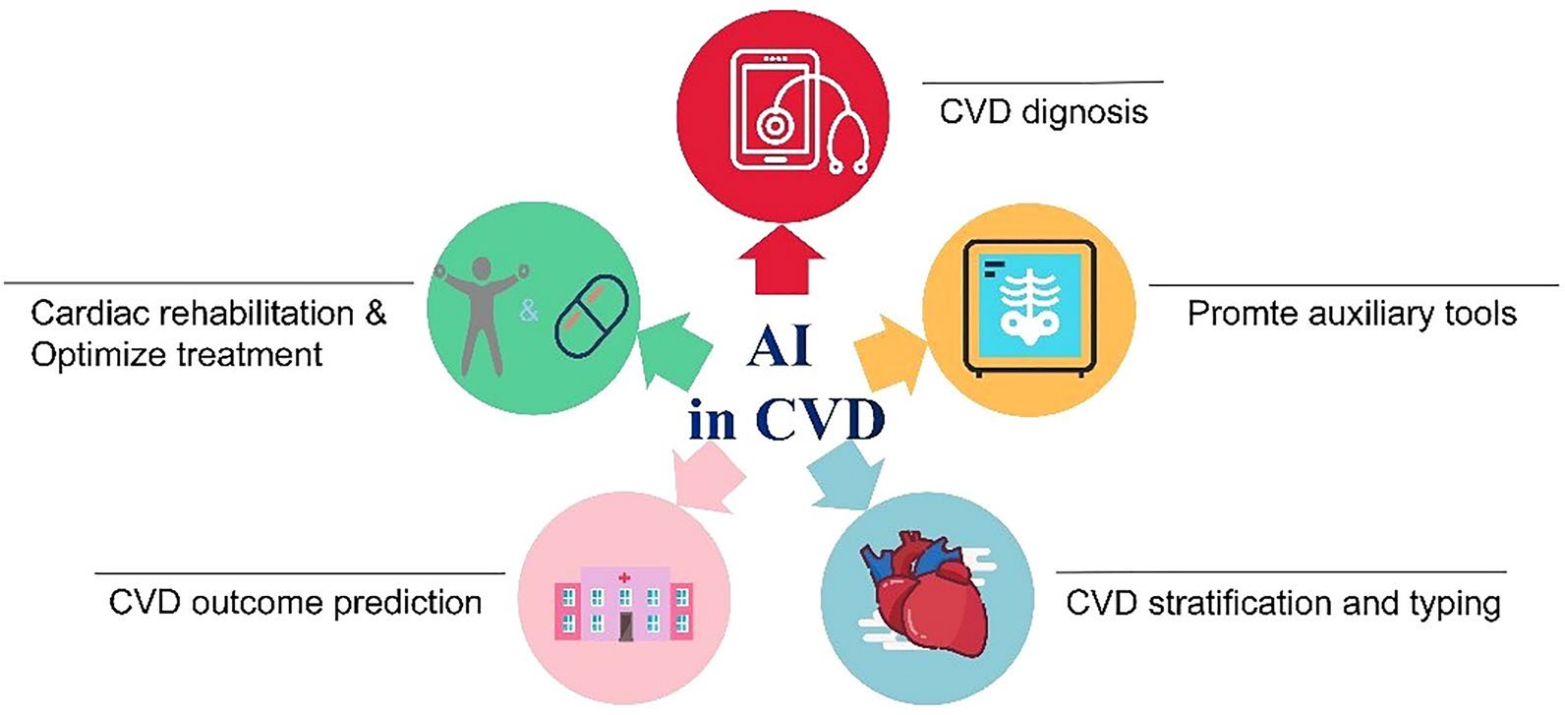
Situation of AI application in CVD

**Fig. 3:**
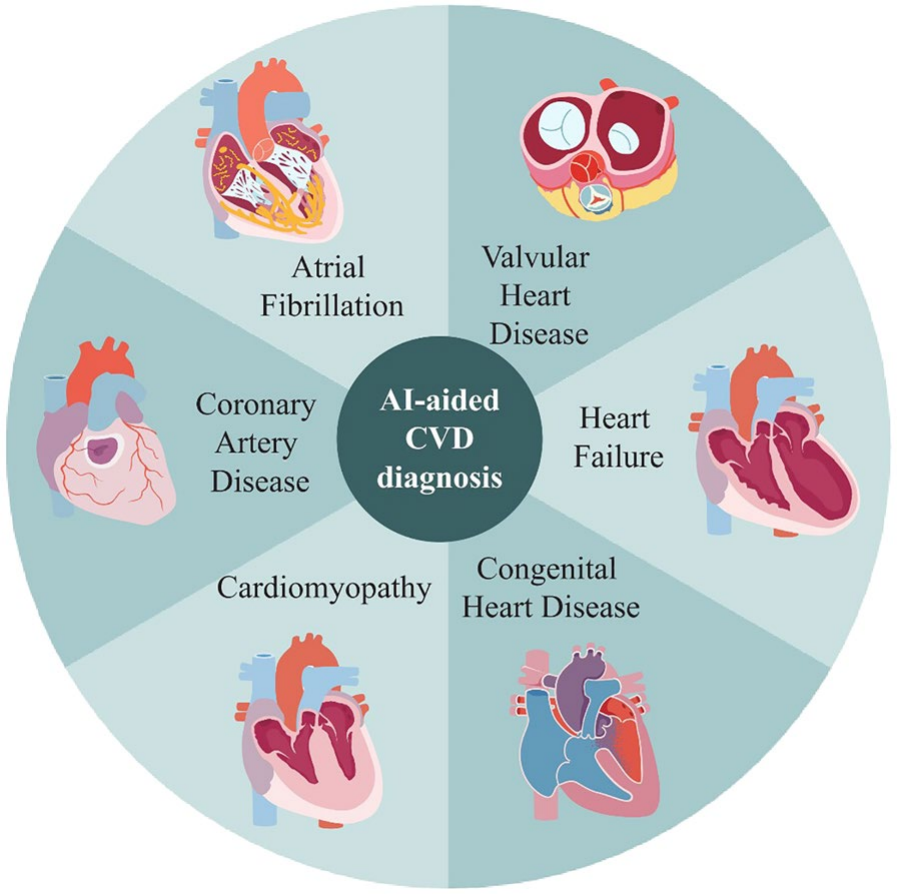
Types of diseases of AI application in CVD

The Receiver Operating Characteristic (ROC) curve and area under the curve (AUC) are essential when quantifying a specified algorithm [23]; in general, it is accepted that an AUC greater than 0.70 has better predictive performance [24]. In cardiovascular medicine, many decisions rely on digitized, patient-specific information, such as ECGs, echocardiograms, and so on, where AI techniques have shown great advantage to benefit patients of different types of CVDs in multiple situations (shown in Figs. 2, 3). This review aims to summarize the application of AI in CVDs through the clinician's perspective to provide clinicians with a better understanding and use of AI.

## AI-aided CVD diagnosis

Early detection, diagnosis, and treatment are significant for CVDs in slowing the progression to advanced diseases and improving overall outcomes. ECG and cardiac magnetic resonance (CMR) are often the gold standards for diagnosing some CVDs, such as ventricular dysfunction, aortic stenosis, and dilated cardiomyopathy [25–28]. However, these additional tools are conducted for patients suspected of having related symptoms rather than for asymptomatic patients [27]. The application of these additional tools is limited by high cost, a requirement for technical expertise that may not be suitable as screening tools for the general population, which brings difficulties for the early diagnosis of CVDs [26, 29]. Therefore, many patients frequently remain undiagnosed until the late stage, with worse outcomes seen in advanced diseases [30].

ECG is a simple, widely available, and low-cost auxiliary test often used even in areas with limited resources [31]. For a long time, ECG has provided valuable diagnostic clues for CVDs. However, the clinician’s interpretation of the ECG depends on the levels of their experience and expertise [31]. Furthermore, the raw ECG waveform contains tens of thousands of data points difficult for clinicians to analyze them, which causes the limitation to exploit its advantage into full use [30]. However, because of its strong computing power, graphic analysis ability, and learning ability, AI can detect subtle and meaningful information from ECG waveforms that clinicians cannot observe, which means the relationship between ECG characteristics and specific CVDs [31, 32]. Here, we will review the latest advances in applying AI technology to routine 12-lead ECG for detecting CVDs in detail.

### Valvular heart disease

Many valvular heart diseases involve long asymptomatic periods [33]. However, once symptoms appear, mortality increases dramatically [34]. Follow-up in asymptomatic patients and valve replacement in symptomatic patients often result in good outcomes [33]. Nevertheless, how to identify these asymptomatic patients remains a challenge. Echocardiography is the gold standard for confirming the diagnosis of valvular heart disease, but it is not suitable for screening tests [35]. Therefore, whether AI-enhanced Electrocardiogram (AI-ECG) can be used as an available tool for screening asymptomatic patients has gained widespread. Kwon et al. [35] developed a DL-based algorithm combining a multilayer perceptron (MLP) and CNN, which aims to detect moderate or severe aortic stenosis (AS) using ECGs. During internal and external validation, the AUC for identifying significant AS were 0.88 and 0.86. Sensitivity analysis showed that the algorithm focused on the T wave of the precordial lead to determine the presence of AS. Interestingly, at the highly sensitive operation point, the negative predictive value was > 99%, suggesting that this algorithm can be used as a screening tool to exclude AS. This work has been confirmed by others. Shelly et al. [36] developed a CNN model for screening moderate or severe AS using ECG and echocardiogram from 129,788 adult patients. AI-ECG performed well in the testing group, including 102,926 participants with an AUC of 0.85 and an accuracy of 74%, and the negative predictive value was 98.9%. When sex and age were added to the model, the AUC was improved to 0.90. Furthermore, elias et al. [30] developed the Valve Net DL model, the AUC for using ECG to detect moderate or severe AS, aortic regurgitation (AR), and mitral regurgitation (MR) was 0.88, 0.77 and 0.83, respectively. The AUC of the composite of any of them was 0.84. Subset analyses showed that the performance of the algorithm was equal in sex, ethnicity, and race. These studies indicate the AI-ECG is a potential measure to screen for valvular heart disease.

### Atrial fibrillation

Atrial fibrillation (AF), especially paroxysmal AF, is often asymptomatic and elusive [37]. Patients with AF often perform a normal sinus rhythm during ECG recording, which may lead to underdiagnosis [38]. However, the structure of the heart starts to change once AF is formed.

Therefore, the normal sinus-rhythm ECGs might undergo subtle changes that a deeply trained neural network can identify to predict AF [6]. Attia et al. [6] have implemented a CNN to identify patients with AF during normal sinus rhythm using standard 10 s, 12-lead ECG. The model was trained using nearly 500,000 ECGs. Applying the model in a testing set yielded an AUC of 0.87 for detecting AF from sinus-rhythm ECGs, with an overall accuracy of 79.4%. When testing the model on all of the ECGs from the patients’ window of interest (from 31 days before the first recorded AF ECG to that day), the AUC improved to 0.90 with improved overall accuracy of 88.3%. The results show that this algorithm can detect patients with AF from normal sinus-rhythm ECG. Afterward, Khurshid et al. [39] compared the accuracy and correlation of AI-ECG and CHARGE-AF (Cohorts for Heart and Aging Research in Genomic Epidemiology-Atrial Fibrillation) scores in predicting future AF risk in three test sets (Massachusetts General Hospital [MGB], Brigham and Women’s Hospital [BWH] and UK Biobank). Over a 5 year follow-up period, AI-ECG provided predictive usefulness to CHARGE-AF in predicting AF. (AUC of MGB, BWH, and UK Biobank were 0.823 vs. 0.802, 0.747 vs. 0.752, and 0.705 vs. 0.732). When the model combined both AI-ECG and CHARGE-AF, it showed better performance on multiple prognosticative model metrics than CHARGE-AF, suggesting that AI-ECG can be used as a useful way to assess the risk of future AF. Besides, AI can also detect future AF by assessing risk factor stratification. In a prospective cohort study, Noseworthy et al. [40] recruited 1003 patients with stroke risk factors but normal sinus-rhythm ECG and divided patients into high-risk or low-risk groups by applying AI algorithm to their ECGs. All participants were then given an ambulatory heart rhythm monitor to detect AF for 30 days. They found that the high-risk group had a higher rate of AF than the low-risk group (7.6% vs. 1.6%). During a median follow-up of 9.9 months, the detection rate of AF was significantly higher in the AI-guided screening group than in the usual care group, suggesting that AI-ECG may be able to identify patients with a high risk of suffering AF in the future. Effective screening of these patients may lead to more effective results.

### Coronary artery disease

Betancur et al. [41] tried to train a DL model to predict future coronary artery disease (CAD) from SPECT myocardial perfusion imaging (MPI). 1638 patients without CAD performed stress SPECT MPI and invasive coronary angiography within 6 months of MPI. The model was evaluated in a stratified tenfold cross-validation procedure. The AUC of CAD prediction was 0.80 per patient and 0.76 per vessel, demonstrating that DL can help the analysis of MPI and predict future CAD. Facial features may be associated with an increased risk of some diseases [42]. DL even enables to screen of diseases by these facial features [43, 44]. Lin et al. [43] trained and validated a DL algorithm to detect CAD using face photos from 5796 patients. In the testing set composed of 1013 patients, the DL algorithm had the AUC of 0.73 and the accuracy of 68% for detecting CAD.

### Heart failure

Left ventricular ejection fraction (LVEF), a key measure of left ventricular systolic function, is often measured by echocardiography [26]. At the early stage of heart failure (HF), patients may present asymptomatic left ventricular dysfunction (ALVD) for a long time because of the slight decrease of LVEF [3, 5]. If patients with HF can get effective treatment, it is very significant to improve their left ventricular systolic functions, avoid the further decline of LVEF and permanent myocardial damage, and improve survival rate and quality of life [45]. However, echocardiography is impractical for asymptomatic patients because of cost and availability [5, 26]. Recently, various studies showed that AI-ECG could be used for screening ALVD. Attia et al. [3] created a large neural network using ECG and echocardiogram of 44,959 patients to identify patients with ventricular dysfunction (ejection fraction [EF] ≤ 35%) by ECG. When tested the network on a set of 52,870 patients, it received the AUC of 0.93, with an accuracy of 85.7%. With a median follow-up of 3.4 years, compared to those who were identified as having a normal EF by both network and echocardiography (i.e., true negative), patients with positive AI-ECG but negative echocardiography (i.e., false positive) had four times more common in developing left ventricular dysfunction (HR = 4.1), suggesting that the network can not only detect patients with left ventricular dysfunction but also identify abnormal ECG before the manifestation of left ventricular dysfunction. Similarly, Yao et al. [5] developed an AI algorithm for identifying patients with low EF (defined as EF ≤ 50%) based on their ECG. 22,641 participants without HF and 120 primary care teams from 45 hospitals were randomly assigned to the intervention or control groups. The intervention group was access to AI-ECG results. This study found that using AI-ECG improved the diagnosis of low EF by 32%, compared to the control group, within 90 days after the ECG test. Furthermore, The AI-ECG improved the diagnosis of low EF more when AI-ECG was applied in the outpatient setting (OR = 1.71), demonstrating that AI-ECG may enable the early diagnosis of patients with low EF in the primary care settings and resource-scarce settings. In contrast, right heart failure is often underreported in the clinical setting, which may be due to the lack of effective screening modalities to evaluate right ventricular function. However, right ventricular dysfunction is closely related to the left and total heart failure. It is urgently needed for an available tool to screen and predict the right hear function. Vaid et al. [26] constructed a DL model that can be used to predict left and right ventricular function from ECG. In the internal and external database, AI-ECG performed well at detecting right ventricular systolic dysfunction (RVSD) with an AUC of 0.84 and detecting patients with LVEF ≤ 40% with an AUC of 0.94, suggesting that the DL model can extract biventricular function information from ECG. AI-ECG can improve the usefulness of screening for left or right ventricular dysfunction.

### Cardiomyopathy

Dilated cardiomyopathy (DC) is a common cause of HF with reduced LVEF. First-degree relatives of patients with DC have an increased risk of DC and are more probably present as sudden death. As a result, these relatives require regular echocardiographic tests [28]. Nevertheless, it is impractical to use echocardiographic to screen asymptomatic populations [29]. Shrivastava et al. [29] built a CNN model to achieve the early diagnosis of DC using ECG. Within a cohort of 421 patients who suffered from DC and 16,025 participants with normal LVEF, the AUC of using AI-ECG to detect LVEF ≤ 45% was 0.955, with a negative predictive value of more than 99%, indicating that AI-ECG may serve as a screening tool for DC and determine the need for subsequent echocardiographic diagnosis among patients. CMR is the gold standard for diagnosing left ventricular hypertrophy (LVH) [25], but limited by its cost and accessibility, it cannot be used as a screening tool for LVH. Khurshid et al. [46] trained a CNN model using ECGs within 32,239 individuals, aiming to predict CMR-tested left ventricular mass based on 12-lead ECG (LVM-AI). When testing in the two independent test sets, LVM-AI predicted LVH with AUC of 0.653 and 0.621, showing that LVM-AI may have a moderate ability to discriminate LVH.

Hypertrophic cardiomyopathy (HCM) is one of the leading causes of sudden cardiac death in young adults [47]. Sudden cardiac death due to HCM is preventable if HCM can be detected early. HCM can be diagnosed with echocardiography, which is widely difficult to use in otherwise asymptomatic individuals [31]. Although more than 90% of patients with HCM have electrocardiogram abnormalities; these are non-specific and indistinguishable from other diseases [48]. AI-ECG may be an effective method to diagnose HCM. Ko et al. [8] trained and validated an AI-ECG with 2448 patients with HCM and 51,153 age- and sex-matched subjects without HCM. When applying this model to diagnose HCM based on ECG in the testing set including 612 patients with HCM and 12,788 control subjects, the AUC was up to 0.96 with the sensitivity of 87% and specificity of 90%. Surprisingly, this model performed particularly well in young individuals (age < 40), suggesting AI-ECG may be practical for screening HCM.

Besides ECG, AI also presents unique advantages in computed tomography (CT) [49], ultrasound [50], SPECT MPI [41], and so on. AI-based DL can even identify some diseases from facial features alone, allowing it to screen many people in a short period [43, 44].

### Congenital heart disease

Congenital heart disease (CHD) is the most common congenital disability, resulting in substantial mortality after birth [51]. In clinical practice, due to a lack of specialized sonographers or missing critical image frames to help the diagnosis of CHD, the detection of CHD during pregnancy is often very low [52]. As AI-ECG, trained AI models can detect abnormal image frames that are difficult for the clinician to discern, improving the diagnosis of CHD [50]. Recently, Arnaout et al. [50] trained a neural network to distinguish normal hearts and CHD using nearly 100,000 images from echocardiographic and screening ultrasound from 18 to 24 weeks. In the internal test set, the model distinguished normal from abnormal hearts with an AUC of 0.99 and achieved a negative predictive value of 100%. Importantly, the model performed robustly on outside-hospital and lower-quality images, suggesting that DL-based screening ultrasound improves the fetal detection of CHD.

AI-based models can be helpful for clinicians to make better decisions [5, 29, 49]. With the popularization of AI technology, AI-based models can help screen diseases and improve the early diagnosis and treatment of diseases in settings with limited equipment [6, 29, 31].

## AI enhances the effectiveness of auxiliary tools

AI techniques could prompt the efficiency of auxiliary tools, such as CT, echocardiography, and magnetic resonance imaging (MRI). LVEF is an essential criterion for the prognosis of HF, resynchronization therapy, and defibrillator. Two-dimensional echocardiography as an accurate tool for assessing EF is widely used in clinical scenarios. In general, quantitative EF requires sophisticated skills to track end-diastolic and end-systolic frames manually, which is a time-consuming process. However, auto EF is the AI-learned pattern that can effectively calculate EF. Asch et al. [53] proposed a ML algorithm that can automate and accurately quantify LVEF, and the ML had similar accuracy to that of clinicians in extracting LVEF. This well-trained advanced algorithm enabled nurses to dynamically observe patients’ LVEF without the professional sonographer, improving clinical safety. Furthermore, the advent of AI created the possibility of monitoring regional wall motion abnormality by screening echocardiograms. Kusunose et al. [54] developed a matching data set of patients with and without a myocardial infraction and trained a deep convolutional neural network (DCNN) to predict the presence of wall motion abnormalities, achieving an AUC of 0.99 similar to cardiologist and sonographer readers (AUC = 0.98) and higher than resident readers (AUC = 0.90).

Coronary computed tomography angiography (CCTA), an extremely effective non-invasive tool, is a first-line inspection method to assess coronary artery stenosis. It is time-consuming, costly, and requires semi-automated manual evaluation. Choi et al. [55] used an AI-based algorithm to enhance CCTA performance by allowing for accurate and rapid assessment of stenosis, atherosclerosis, and vessel morphology compared with the consensus of expert readers at level 3. Lin et al. [56] trained a DL pattern to quantify plaque and stenosis using coronary CT angiography. Besides, this DL algorithm might have diagnostic significance in predicting prognosis of myocardial infarction. In addition, Knott et al. [57] used AI algorithms to quantify myocardial blood flow (MBP) and myocardial perfusion reserve (MPR) by CMR and evaluate the algorithms in a cohort study of 1049 patients with 605 median follow-up days. The results enrolled in a Cox proportional hazard model and deduced that reduced MBF and MPR used AI-based quantified CMR provided an independent predictor of cardiac prognosis.

Although, invasive fractional flow reserve (FFR) is a gold criterion for diagnosing coronary stenosis, over 70% clinical treatment decisions still rely on angiography. However, angiography has lacking accuracy for the prediction stenosis for the prediction of FFR < 0.80. Hae et al. [58] have found that machine learning facilitates bridging the visual- functional mismatch between FFR and angiography. Not only that, Cho et al. [59] performed a ML-based model trained by extreme gradient boosting (XGBoost) could accurately predict samples with FFR ≤ 0.80, achieving an AUC of 0.84.

## AI-aided CVD stratification and typing

Novel approaches based on AI are able to provide more accurate stratification and typing for CVD patients, which might be a way to overcome some of the limitations of traditional approaches and optimize personalized medicine. One area where more accurate phenogrouping could improve the selection of patients is cardiac resynchronization therapy (CRT), since the traditional strategy did not work out well and a substantial proportion of patients do not respond to this therapy. Cikes et al. [60] trained an unsupervised ML algorithm to categorize subjects by similarities in clinical parameters, left ventricular volume, and deformation traces at baseline into four exclusive groups. Finally, four phenogroups were identified and two phenogroups were associated with a substantially better treatment effect of CRT with a defibrillator vs. implantable cardioverter defibrillator than observed. Similarly, measures remained limited in patients with HF and reduced LVEF despite advances in therapeutics. Karwath et al. [61] applied neural network-based variational autoencoders and hierarchical clustering to pooled individual patient data from nine double-blind, randomised, placebo-controlled trials of β blockers. The AI-based clustering approach was able to distinguish prognostic response from β-blockers both in sinus rhythm patients as well as patients with concomitant AF.

AF is a cardiovascular condition which has a multifactorial origin. Epidemiological data clearly demonstrates that the concomitant presence of multiple risk factors increases the risk of developing AF [62]. Proietti et al. [63] performed a hierarchical cluster analysis derived from EORP-AF (the European Society of Cardiology-European Heart Rhythm Association EURObservational Research Programme in AF) General LongTerm Registry and identified three clusters. Over a mean follow-up of 22.5 months, Cluster 3 had the highest rate of cardiovascular events, all-cause death, and the composite outcome (combining the previous two) compared to Cluster 1 and Cluster 2, suggesting that cluster analysis might be a choice for providing information of AF patients’ clinical phenotypes and prognostic events.

Damping of aortic pressure during coronary angiography can help avoid serious complications and make accurate coronary physiology measurements. To accurately identify damped arterial waveform traces in real-time during invasive coronary angiography, Howard et al. [64] trained a 1-dimensional CNN on two pre-existing data sets from patients undergoing invasive coronary angiography at 4 European cardiac centers. The model can classify beats as either normal, showing damping, or artifactual with 99.4% accuracy when judged against the opinions of the internal core laboratory and 98.7% accuracy when judged against the opinions of an external core laboratory not involved in neural network training.

The phenotypic features of hypertensive patients are highly heterogeneous in cardiovascular outcomes and comorbidities, as well as responses to antihypertensive pharmacotherapy. Therefore, it is of critical importance to classify hypertensive patients with clinically meaningful labels, optimize management strategies and forecast prognostic trajectory. Yang et al. [65] performed an unsupervised, data-driven cluster analysis on all baseline variables related to cardiovascular outcomes and treatment responses in subjects from the Systolic Blood Pressure Intervention T trial (SPRINT) and identified four replicable clusters. Cluster 4 had the highest risk of developing primary CVD outcome. Intensive antihypertensive treatment was shown to be beneficial only in cluster 4 and cluster 1 and was associated with an increased risk of severe adverse effects in cluster 2. Except for primary hypertension (PHT), endocrine hypertension (EHT) is frequently overlooked or misdiagnosed as PHT in current clinical scenarios. Reel et al. [66] used a supervised ML algorithm to distinguish different EHT subtypes from PHT by multi-omics (MOmics) feature, including plasma miRNAs, plasma catechol O-methylated metabolites, plasma steroids, urinary steroid metabolites, and small plasma metabolites. Among six ML models, the random forest model performed the best distinguishing primary aldosteronism (PA), pheochromocytoma/catecholamine-producing paraganglioma (PPGL), and Cushing’s syndrome (CS) and PHT (AUC = 0.95) with 57 MOmics features. For discrimination of EHT (PA + PPGL + CS) vs. PHT, the simple logistic classifier achieved 0.96 AUC with 37 MOmics features.

In up to 60% of HCM patients, more than 1400 mutations in genes encoding sarcomere proteins have been detected [67]. The selection of patients with a high probability of positive HCM genotypes can maximize the cost-effectiveness of genetic testing. Zhou et al. [68] trained a nonenhanced cine CMR image–based DL model to explore the potential value of CMR in reflecting HCM genotype status. Model performance was assessed using a tenfold cross-validation on the internal data set with an AUC of 0.80 and an accuracy of 78.43%. In addition, the combination of the DL model and the Toronto score (with an AUC of 0.84 and an accuracy of 84.31%) yielded significantly higher diagnostic performance than a single score.

## AI-aided CVD outcome prediction

AI-based prognostic models are widely developed in cardiovascular medicine. Recent advances in the application of AI for standard 12-lead ECG enable prediction of long-term outcomes for CVD patients. To develop and validate a DNN model to predict 1 year all-cause mortality from ECG voltage-time traces, Raghunath et al. [69] used 1,169,662 12-lead resting ECGs obtained from 253,397 patients over a 34 year period in a large regional health system. Even within the large subset of patients (*n* = 45,285) with ECGs interpreted as ‘normal’ by a physician, the performance of the model in predicting 1 year mortality remained high (AUC = 0.85), indicating that AI can add substantial prognostic information to the interpretation of ECG. Besides, ECG-derived age by AI might serve as a way to predict cardiovascular events. Toya et al. [70] revealed that there was a difference between AI-estimated age from ECG and chronological age, which was defined as Δ age. By accessing peripheral microvascular endothelial function (PMEF), an indicator of vascular aging, the authors found that Δ age was higher in patients with abnormal PMEF than in patients with normal PMEF. Furthermore, patients with abnormal PMEF and higher Δ age have a marked increase in risk for major adverse cardiovascular events (MACE).

Substantial prospective epidemiological data have demonstrated that changes in retinal-vessel caliber are associated with classic CVD risk factors. However, most software for estimation is semi-automated, requiring human intervention to adequately measure retinal-vessel caliber on the basis of prespecified protocols. Cheung et al. [71] trained a CNN model to automatedly access retinal-vessel caliber in retinal photographs based on diverse multiethnic multicountry data sets that comprise more than 70,000 images. In conclusion, the CNN model was able to accurately access CVD risk factors comparably to or better than expert graders, providing the possibility of clinical application of end-to-end DL systems for the prediction of CVD events on the basis of the features of retinal vessels in retinal photographs.

AI-based models have been proven to perform well in prognostic assessment in CAD patients. Silva et al. [72] used the survival tree (ST) algorithm to predict the morality of CAD patients referred to a cardiac rehabilitation program in a retrospective cohort linking clinical, administrative, and vital status databases from 1995 to 2016. Age and peak metabolic equivalents (METs) were chosen as the features with the greatest importance for mortality prediction, using which ST split patients into 8 clusters with different survival probabilities (*P* < 0.001). Backhaus et al. [73] compared AI automated with manual assessments for left ventricular function assessment, included global longitudinal, circumferential, and radial strains (GLS/GCS/GRS) from prospectively recruited acute myocardial infarction (AMI) populations. GLS showed the best and excellent agreement with an intraclass correlation coefficient (ICC) of 0.81 and was an independent predictor of MACE in multivariate analyses (HR 1.10, 95% CI 1.04–1.15, *P* < 0.001).

Coronary artery calcium is an accurate predictor of cardiovascular events. While it is visible on all CT scans of the chest, this information is not routinely quantified as it requires expertise, time, and specialized equipment. Zeleznik et al. [74] suggested a DCNN model trained on a data set from 20,084 individuals from distinct asymptomatic (Framingham Heart Study, NLST) and stable and acute chest pain (PROMISE, ROMICAT-II) cohorts. The study showed that the automated score is a strong predictor of cardiovascular events, independent of risk factors (multivariable-adjusted HR = 4.3), and shows a high correlation with manual quantification and robust test-retest reliability. Epicardial adipose tissue (EAT) volume and attenuation (Hounsfield units) may predict MACE. Eisenberg et al. [75] used a fully automated DL algorithm to qualify EAT volume and attenuation from non-contrast cardiac computed tomography of 2068 asymptomatic subjects without known CAD enrolled in the EISNER trial. The participants completed long-term (over 14 years) prognostic follow-ups. The study revealed that DL-based EAT was associated with increased risk of MACE, and EAT attenuation was inversely associated with MACE.

Intravascular ultrasound (IVUS) is a useful tool for planning percutaneous coronary intervention (PCI) by providing information on lesion characteristics, vessel size, and suboptimal stent deployment. Min et al. [76] used a pre-procedural IVUS-based CNN and XGBoost model to predict the occurrence of the stent under expansion. A total of 618 coronary lesions were randomized into training and test sets in a 5:1 ratio, and the model performed well with an accuracy of maximal accuracy of 94% (AUC = 0.94).

Most clinical risk stratification models are based on measurement at a single time-point, which always provides limited information [77]. However, AI enables the use of multi-dimensional data, such as changes of variables over time, and serves as a more accurate prediction. Vitamin K antagonists (VKAs) are prescribed to prevent stroke in AF patients. Clinicians adjust the dose of VKA based on an individual patient's prothrombin time international normalized ratio (PT-INR) at each visit [78]. Goto et al. [79] suggested an RNN trained with multi-dimensional patient-level PT-INR values obtained within the first 30 days after starting treatment. Patients experienced a follow-up over 31–365 days after enrolment. The model performed better than time in therapeutic range (TTR) at predicting clinical outcomes occurring up to 12 months thereafter.

AI is also a new technological approach to improve the accuracy of risk prediction before and after cardiac surgery. Kilic et al. [80] suggested an XGBoost ML algorithm from 243,142 adult patients undergoing isolated surgical aortic valve replacement (SAVR) in the Society of Thoracic Surgeons (STS) National Database between 2007 and 2017, which were randomly split 4:1 into training and validation sets. In conclusion, the model demonstrated excellent calibration and modest improvements compared with existing STS models in predicting outcomes of SAVR. Hospital readmission has the interest of researchers due to its adverse impacts on healthcare budgets and patient loads. To predict 30 day hospital readmission after cardiac surgery, Sherman et al. [81] chose predictors including demographics, preoperative comorbidities, proxies for intraoperative risk, indicators of postoperative complications, and time series-derived variables to training several machine learning models and evaluated each on a held-out test set. A random forest model performed best, which worked out to have a mean AUC of 0.76.

## Limitations

However, several issues need to be solved before AI technology can be used in auxiliary diagnosis: (1) Due to humans cannot comprehend the intermediate layers of AI network, most studies have not clarified what gives a model its ability to detect diseases, the explanation of which needs to be further studied to enhance user trust of AI tools [6, 29, 32, 35]; (2) Existing conclusions are based on only several research sites and their patients, continued studies would be required to confirm the reliability of these models on a larger scale and more patients [26, 29, 35]. (3) Further studies are needed to determine the cost-effectiveness of AI technology in auxiliary diagnosis and estimate their impact on clinical practice [5, 32].

## Data Availability

The raw data supporting the conclusions of this article will be made available by the authors, without undue reservation.
